# *In silico* screening and identification of CTL and HTL epitopes in the secreted virulence factors of Mycobacterium tuberculosis

**DOI:** 10.5114/bta/201461

**Published:** 2025-03-31

**Authors:** Edward Kevin B. Bragais, Francisco M. Heralde, Kim Claudette J. Fernandez, Salvador Eugenio C. Caoili, Leana Rich Herrera-Ong

**Affiliations:** 1 Department of Biochemistry and Molecular Biology, College of Medicine, University of the Philippines Manila, Manila, Philippines; 2 Department of Biology, School of Science and Engineering, Ateneo De Manila University, Quezon, Philippines

**Keywords:** *Mycobacterium tuberculosis*, cytotoxic T-cell epitopes, helper T-cell epitopes, immunoin-formatics

## Abstract

**Background:**

*Mycobacterium tuberculosis* (MTb) is a highly infectious pathogen and a global health threat due to its resilient cell wall and immune evasion strategies. Despite the availability of the antituberculosis Bacille Calmette-Guérin (BCG) vaccine, its efficacy varies (0%–80%) and gradually decreases over time. This study aimed to identify cytotoxic T-lymphocyte (CTL) and helper T-lymphocyte (HTL) epitopes in MTb secretory proteins using immunoinformatics tools.

**Materials and methods:**

The Protein Variability Server was used to identify highly conserved sequences, and epitope population coverage was estimated for the Southeast Asian (SEA) region. Selected epitopes were also docked to their major histocompatibility complex alleles.

**Results:**

Five secretory proteins critical to MTb pathogenesis and virulence were identified as antigenic (antigenicity score > 0.4). Predicted epitopes had IC_50_ values ≤ 500 nM, indicating strong binding affinity, with an estimated 94% population coverage in SEA. All candidate epitopes were highly conserved (Shannon index ≤ 0.1) and showed no significant sequence similarity to human proteins, allergens, or toxic peptides. Docking analysis confirmed favorable binding to their corresponding HLA alleles, as indicated by low Gibbs free energy change (Δ*G*) values and dissociation constants (*K*_D_ nM).

**Conclusions:**

Overall, this study identified immunoactive CTL and HTL epitopes that could serve as promising candidates for future antiTB vaccine development. Further *in vitro* and *in vivo* studies are required to validate these preliminary findings.

## Introduction

Tuberculosis (TB) is a highly contagious bacterial pathogen caused by *Mycobacterium tuberculosis* (MTb) (Smith, 2003). According to the World Health Organization (WHO), TB remains one of the leading global causes of death, with 1.6 million fatalities and 10.6 million new cases reported in 2021 (WHO, 2023). The Philippines ranks as the fourth-highest contributor to TB cases worldwide (Montemayor, 2023). The pathology of TB results from a dynamic interaction between the host immune system and the bacterium. MTb is transmitted through airborne droplets from the coughs and sneezes of an infected individual. Upon inhalation of these droplets containing MTb, the bacilli will colonize the primary site of infection, the lungs (pulmonary), and spread throughout the body (extrapulmonary), particularly those immuno-compromised (WHO, 2023).

Current TB treatment involves a combination of anti-biotics and vaccination; however, eradication efforts are increasingly challenged by the emergence of multidrug-resistant MTb strains. Morphological analyses have shown distinct differences between active and latent TB infections (Alva et al., 2013). The formation of granuloma is one of the pathological hallmarks of active TB infection. The granuloma is formed from the organized packing of immune cells such as macrophages, lymphocytes, and multinucleated giant cells, which is the host’s attempt to confine and control the spread of MTb. Moreover, the granuloma may undergo caseous necrosis, characterized by a cheese-like, necrotic material, due to the breakdown and death of macrophages infected with MTb. On the other hand, latent TB infection does not manifest caseous necrosis. Instead, the granuloma may contain viable MTb cells in either a dormant or non-replicating state (Ehlers and Schaible, 2013; Pal et al., 2022).

Recent advancements in proteomic technologies have identified proteins involved in MTb infection, revealing complex host–pathogen interactions and potential therapeutic targets (Yimer et al., 2017). Several well-characterized MTb proteins are implicated in immune evasion, nutrient acquisition, cell wall biosynthesis, and virulence. Among these, secretory proteins have gained particular scientific due to their ability to modulate and evade immune responses effectively (Pal et al., 2022; Upadhyay et al., 2018).

Secretory proteins are proteins involved in the exportation or secretion of proteins beyond the cytoplasmic membrane and, oftentimes, outside the cell of many bacteria, including mycobacteria (Pal et al., 2022). However, species under the genus *Mycobacterium* use these secretory proteins as a protein export system for their virulence and pathogenesis. The specialized protein secretion system or ESX system (early secretory antigenic target (ESAT-6) secretion system) is considered vital for the virulence and survival of mycobacteria (Houben et al., 2014). The genus *Mycobacterium* has five specialized ESX export systems (ESX-1 to ESX-5), collectively known as the *Type VII secretion system*. Secretory proteins within the ESX system share three unique characteristics: (1) they lack signal peptides, (2) their secretion depends entirely on the ESX system, and (3) they contain a highly conserved Trp-X-Gly (WXG) motif essential to their function (Abdallah et al., 2007; Pallen, 2002).

MTb secretory proteins manipulate host immune responses to establish infection through various mechanisms. These proteins are either secreted within the phagosome – where they can leak into the cytoplasm – or directly into the cytoplasm, where MTb occasionally localizes (Sreejit et al., 2014; van der Wel et al., 2007). Some secretory proteins exhibit kinase and phosphatase activities that interfere with host signaling molecules (Pradhan et al., 2018; Tiwari et al., 2009), while others possess unique eukaryotic nuclear localization signals (NLS) that enable translocation to the host nucleus, where they interact with chromatin and disrupt transcription (Bhat et al., 2017; Sreejit et al., 2014; Yaseen et al., 2015). Additionally, certain secretory proteins inhibit phagosome maturation and phagosome–lysosome fusion, preventing the host immune system from countering the bacterial infection. Key examples include *ESAT-6*, *outer membrane channel protein* (CpnT), *ESX-1 secretion-associated protein* (EspG1), *serine/threonineprotein kinase* (PknG), and *sphingomyelinase* (SpmT) (Pal et al., 2022).

Identifying MTb virulence factors, particularly secretory proteins involved in immune modulation, is a crucial step toward developing more effective vaccines (Kroesen et al., 2019). Vaccination remains a primary preventive measure against TB, but the currently available Bacille Calmette-Guérin (BCG) vaccine has significant limitations, providing inconsistent protection in children and adults (Bolz et al., 2016; Zimmerman et al., 2018). Immunogenic epitopes may provide solutions for the limited protection and associated side effects of live attenuated vaccines among immunocompromised individuals. Prediction of immunogenic peptide epitopes using the immunoinformatics approach is an effective technique that has proven cost-effective and time-efficient (Oli et al., 2020). Thus, this study aimed to identify cytotoxic (CTL) and helper T lymphocyte (HTL) epitopes from well-characterized MTb virulence factors using various immunoinformatics tools and databases. The researchers identified potentially immunoactive epitopes with broad population coverage and minimal adverse reactions, which could serve as promising components of next-generation multiepitope-based vaccines against MTb.

## Materials and methods

### Identification and retrieval of protein sequences

Five MTb secretory proteins – early secretory antigenic protein 6 kDa (ESAT-6), sphingomyelinase (SpmT), outer membrane channel protein (CpnT), ESX-1 secretion-associated protein (EspG), and serine/threonine protein kinase (PknG) — were selected based on their established roles in MTb pathogenesis.

To finalize the list of candidate secretory proteins (antigens), the antigenicity of each protein was assessed using VaxiJen v.2.0 (https://www.ddg-pharmfac.net/vaxijen/VaxiJen/VaxiJen.html), with a bacterial model threshold set at ≥ 0.4. VaxiJen v.2.0 has a reported accuracy of approximately 70–89% (Doytchinova and Flower, 2007). Antigenicity predictions were further validated using ANTIGENpro, which has a reported accuracy of 76% (Magnan et al., 2010).

The protein sequences of the selected secretory proteins were retrieved from the National Center for Biotechnology Information (NCBI) protein database (https://www.ncbi.nlm.nih.gov/protein). Five sets of retrieved protein sequences for each antigen were subjected to sequence alignment using Clustal Omega (https://www.ebi.ac.uk/Tools/msa/clustalo) to assess sequence conservation.

### Identification of highly conserved protein sequences

Highly conserved sequences of the five selected MTb secretory proteins were identified using the Protein Variability Server (PVS) (http://imed.med.ucm.es/PVS/) tool. The consensus sequences generated by PVS were used as input for further analyses.

To ensure sequence conservation, peptide fragments containing at least nine amino acid residues were collected. Amino acid residues with a Shannon index greater than or equal to 0.1 (*S* ≥ 0.1) were masked and excluded from further analysis. The resulting peptide fragments were summarized according to their respective amino acid residue positions in the sequence (Garcia-Boronat et al., 2008).

### Prediction of cytotoxic and helper T lymphocyte epitopes

CTL and HTL epitopes were predicted from the highly conserved sequences of ESAT-6, SpmT, CpnT, EspG, and PknG using immunoinformatics tools available in the Immune Epitope Database (IEDB) (http://tools.iedb.org/processing). Default thresholds in IEDB were applied for epitope prediction. For CTL epitope prediction, the Proteasomal Cleavage/TAP Transport/MHC Class I Combined Predictor was used, specifically employing the netMHCcons algorithm. This method integrates multiple MHC-peptide binding prediction algorithms, including netMHCpan, PickPocket, and netMHC (ANN), to enhance prediction reliability and accuracy (Karosiene et al., 2012). Thirty-six of the most common HLA class I alleles were included for CTL epitope predictions (Paul et al., 2013). Default parameters were used for: Proteasomal cleavage prediction (*immunoproteasome* model); and TAP transport prediction (*maximum precursor extension = 1; alpha factor = 2*). The prediction results were tabulated, including each peptide’s proteasome score, TAP score, and MHC-peptide binding score. Candidate CTL epitopes were selected based on the following criteria: Positive proteasome and TAP scores; MHC IC_50_ scores ≤ 500 nM (indicating strong binding affinity); Binding promiscuity with at least two HLA class I alleles. Peptides meeting these criteria were included in the final set of candidate CTL epitopes for further analysis.

The same highly conserved peptide fragments were used as input for predicting HTL epitopes using default thresholds in the IEDB platform. Prediction methods included netMHCII-2.3, NetMHCIIPan 4.1 EL, and Consensus 2.22, with epitope binding assessed against 27 commonly expressed HLA class II alleles (Greenbaum et al., 2011). The consensus HTL epitopes were selected based on MHC IC_50_ scores (IC_50_ ≤ 500 nM) and promiscuous binding with at least two HLA class II alleles.

The Universal Protein Resource (UniProt) server (https://www.uniprot.org) was used to search for information on the post-translationally modified sites (glycosylation, phosphorylation, etc.) of the five secretory proteins. Epitopes containing amino acid residues that were found to be post-translationally modified were discarded.

### Estimation of population coverage and evaluation of potential cross-reactivity, allergenicity, and toxicity

The population coverage of the predicted CTL and HTL epitopes for Southeast Asia was estimated using the Population Coverage tool in IEDB (http://tools.iedb.org/population/), where the coverage for each candidate epitope per secretory protein was calculated based on its corresponding HLA allele binders. To assess potential cross-reactivity, the Protein Basic Local Alignment Search Tool (BLASTp) in NCBI (https://blast.ncbi.nlm.nih.gov.ph/BLAST.cgi?PAGE=Proteins) was used to compare the predicted epitopes against available human (*Homo sapiens*) protein sequences in the protein databank (PDB), excluding uncultured, model, and environmental sample sequences. Additionally, AllergenFP v.1.0 (https://ddg-pharmfac.net/AllergenFP/) was used to predict the probable allergenicity of the candidate epitopes, employing the Tanimoto score to compare the sequences with known allergens, with a reported prediction accuracy of 80% (Dimitrov et al., 2014). Potential toxicity was further assessed using ToxinPred (https://webs.iiitd.edu.in/raghava/toxinpred/), which applies default SVM parameters and has an estimated accuracy of 90% (Gupta et al., 2013).

### Molecular docking analysis of the candidate epitopes

To support the results obtained from IEDB, molecular docking analysis was conducted for the candidate CTL and HTL epitopes. The top 10 epitopes with the highest population coverage from each secretory protein were selected for further analysis, and HLA binders with the highest IC_50_ were used for docking visualization and binding affinity analysis. Crystal structures of HLA class I and II alleles were retrieved from protein structure databases, and peptides originally bound to the HLA crystal structures were removed to prepare for docking using GalaxyPepDock (https://galaxy.seoklab.org/cgi-bin/submit.cgi?type=PEPDOCK). Unrelated heterostructures were also removed during preliminary complex preparation (Hernandez-Santoyo et al., 2013). The docked and energy-optimized structures of the HLA-epitope complexes with the highest Template Modelling (TM) scores were retrieved from the GalaxyPepDock server. Visualization and structural exploration of each complex were conducted using ChimeraX v.1.3, while binding affinity, represented by Gibbs free energy change (Δ*G*) and dissociation constant (*K*_D_), was estimated in the PRODIGY server (https://wenmr.science.uu.nl/prodigy/)at an average human body temperature of 37°C. These Δ*G* and *K*_D_ values were then compared with positive reference HLA-epitope complex structures available in the database.

## Results

### Antigenicity of the five secretory proteins of MTb

The retrieved protein sequences of the five secretory proteins of MTb were classified as antigenic based on their antigenicity scores, which ranged from 0.4378 to 0.5577 using VaxiJen v.2.0. This classification was further supported by ANTIGENpro, where the predicted probability of antigenicity ranged from 0.4330 to 0.9069. The data is summarized in Table 1.

**Table 1:** Antigenicity assessment of five secretory Mycobacterium tuberculosis proteins

Secretory protein	Antigenicity score (Vaxijen)	Antigenicity score (ANTIGENpro)
ESAT-6	0.5577	0.9069
SpmT	0.5372	0.4726
CpnT	0.5097	0.6027
EspG	0.4378	0.6783
PknG	0.4952	0.4330

### Highly conserved sequences from the five secretory proteins of MTb

Highly conserved fragments from the sequences of the five MTb secretory proteins exhibited Shannon variability indices ≤ 0.1. The length of fragments ranges from 12 to 299 amino acid residues exhibiting minimal variability in these five secretory proteins across various strains of MTb. The summary of the highly conserved sequences can be accessed in the supplementary material.

### CTL and HTL epitopes mapped from the highly conserved sequences

All candidate CTL epitopes mapped from the highly conserved sequences of the five secretory proteins exhibited positive TAP and proteasome scores and IC_50_ values ≤ 500 nM, indicating strong binding affinity to HLA class I alleles (Fleri et al., 2017). Additionally, each candidate CTL epitope demonstrated promiscuous binding to at least two HLA class I alleles, increasing the likelihood of broader population coverage (Bhasin and Raghava, 2007). Similarly, all HTL epitopes included in the final selection exhibited good binding affinity to at least two HLA class II alleles, with IC_50_ values ≤ 500 nM. The total number of finalized candidate CTL and HTL epitopes is summarized in Table 2, while the masked sequences of all predicted candidate epitopes from the five secretory proteins can be accessed in the supplementary material.

**Table 2:** Summary of the total number of cytotoxic and helper T lymphocyte classified as nonallergenic, nontoxic, and non-cross-reactive epitopes

Secretory protein	CTL predicted epitopes	HTL predicted epitopes
ESAT-6	32	26
SpmT	41	95
CpnT	147	109
EspG	81	138
PknG	99	230

CTL – cytotoxic T lymphocyte, HTL – helper T lymphocyte

### Population coverage of the CTL and HTL epitopes

Population coverage analysis was performed in IEDB for both individual and complete sets of CTL and HTL epitopes. Results showed that the set of CTL epitopes for each secretory protein has population coverage ranging from 87.88% to 96.82%, 76.47% to 94.46%, and 91.46% to 99.24%, for the Philippines, the Southeast Asian region, and the world, respectively. On the other hand, the population coverage of the set of HTL candidate epitopes of the five secretory proteins has population coverage ranging from 14.88% to 27.53, 32.8% to 53.81%, and 44.22% to 78.76%, for the Philippines, the Southeast Asian region, and the world, respectively. The data is summarized in Table 3, while the top 10 candidate epitopes with the highest population coverage for each secretory protein are available in the supplementary files.

**Table 3:** Summary of the population coverage of candidate cytotoxic (CTL) and helper (HTL) T lymphocyte epitopes

Secretory protein	Philippines		Southeast Asia		World	
	CTL	HTL	CTL	HTL	CTL	HTL
ESAT-6	87.88%	23.42%	81.74%	34.97%	91.46%	44.22%
SpmT	90.64%	25.52%	76.47%	48.11%	93.75%	71.90%
CpnT	98.10%	14.88%	94.46%	32.8%	99.24%	47.57%
EspG	90.95%	27.53%	85.82%	53.81%	97.09%	78.76%
PknG	96.82%	26.57%	93.38%	51.48%	98.60%	72.95%

### Evaluation of potential cross-reactivity, allergenicity, and toxicity

Further analysis of cross-reactivity, allergenicity, and toxicity confirmed that all candidate CTL and HTL epitopes are noncross-reactive, nonallergenic, and non-toxic, based on established parameters, thresholds, and similarity scores across different predictive tools. The *E*-value of the epitopes ranged from 3.3 to 85, which indicates that none of the epitopes were cross-reactive.

### Molecular docking between HLA alleles and selected T lymphocyte epitopes

**Figure 1:**
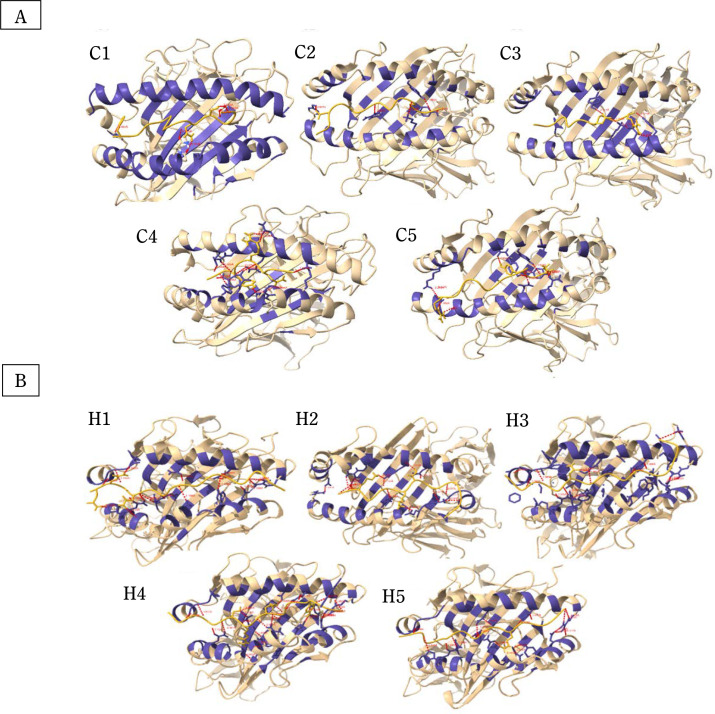
Molecular docking analysis and visualization of some (**A**) cytotoxic (C1–C5) and (**B**) helper (H1–H5) T limphocyte epitopes docked within the binding pockets of their corresponding HLA structure showing the hydrogen bond interactions (red-dotted lines)

Figure 1 shows the structures of CTL and HTL epitopes docked within the peptide binding groove of their corresponding HLA alleles. The formation of the HLA-epitope complex is favorable, as indicated by a negative Gibbs free energy change and low dissociation constant values (Table 4). These values confirm that all epitopes are good to strong binders of their respective HLA alleles (Koyanagi et al., 2010; Paul et al., 2013).

**Table 4:** Summary of the dissociation constant and binding energy of the HLA–epitope complexes

Secretory protein	T-cell subtype	HLA-epitope complex	ΔG (kcal/mole)	KD (mol/l)
ESAT-6	CTL	C1: HLA-A*11:01-HSLLDEGKQSLTK	*–*16.4	2.60 × 10^–12^
	HTL	H1: HLA-DRB1*04:01-QQWNFAGIEAAASAI	*–*16.8	1.50 × 10^–12^
SpmT	CTL	C2: HLA-A*02:06-LTLKGFTY	*–*16.6	2.10 × 10^–12^
	HTL	H2: HLA-DRB1*15:01-KRLNAYYVANVQEDF	*–*21.9	3.40 × 10^–16^
CpnT	CTL	C3: HLA-A*02:06-RYQERFNSV	*–*16.4	2.70 × 10^–12^
	HTL	H3: HLA-DRB1*01:01-RFLHSNPVGVVIDGTG	*–*21.4	8.50 × 10^–16^
EspG	CTL	C4: HLA-A*11:01-AVYARQYR	*–*16.1	4.50 × 10^–12^
	HTL	H4: HLA-DRB1*01:01-VVALLSRGKLLYGVI	*–*24.8	3.40 × 10^–18^
PknG	CTL	C5: HLA-A*11:01-KTYDSYGRLLR	*–*16.8	1.50 × 10^–18^
	HTL	H5: HLA-DRB1*01:01-GQSLKRSKGQKLPV	*–*21.5	6.80 × 10^–16^

Additionally, 3D visualization of the HLA-epitope complex (Figure 1) revealed that most intermolecular interactions involved hydrogen bonding between the HLA allele’s peptide-binding groove and the epitope. It is well-established that hydrogen bonds contribute significantly to the stability and strength of protein–ligand interactions (Chen et al., 2016; Paul et al., 2013). Detailed information on the number of hydrogen bonds and the donor–acceptor hydrogen bond distance range is provided in Table 5.

**Table 5:** Protein–peptide interactions in the HLA–epitope complex

Secretory protein	T-cell subtype	HLA–epitope complex range	No. of H-bonds	Donor–acceptor hydrogen distance (Å)	Donor–acceptor distance range (Å)
ESAT-6	CTL	C1: HLA-A*11:01-HSLLDEGKQSLTK	6	2.054–2.404	2.950–3.261
	HTL	H1: HLA-DRB1*04:01-QQWNFAGIEAAASAI	10	1.884–2.419	2.839–3.091
SpmT	CTL	C2: HLA-A*02:06-LTLKGFTY	5	1.910–2.054	2.717–3.015
	HTL	H2: HLA-DRB1*15:01-KRLNAYYVANVQEDF	15	1.889–3.068	2.712–3.694
CpnT	CTL	C3: HLA-A*02:06-RYQERFNSV	5	1.901–2.319	2.800–3.052
	HTL	H3: HLA-DRB1*01:01-RFLHSNPVGVVIDGTG	14	1.885–2.678	2.781–3.658
EspG	CTL	C4: HLA-A*11:01-AVYARQYR	12	1.868–2.582	2.722–3.328
	HTL	H4: HLA-DRB1*01:01-VVALLSRGKLLYGVI	27	1.826–2.664	2.666–3.232
PknG	CTL	C5: HLA-A*11:01-KTYDSYGRLLR	10	1.884–2.419	2.839–3.091
	HTL	H5: HLA-DRB1*01:01-GQSLKRSKGQKLPV	19	1.880–2.376	2.778–3.538

## Discussion

MTb is recognized as one of the most successful bacterial pathogens in human history (Pal et al., 2022). It is the causative agent of TB, a life-threatening disease that continues to pose a global health challenge. Despite the availability of antibiotics and vaccines, current treatment strategies provide limited protection, particularly for children, adults, and immunocompromised patients — groups that account for a significant proportion of TB cases worldwide (Riccomi et al., 2019).

The first-line treatment for TB typically involves a combination of antibiotics, including isoniazid and rifampicin (Prasad et al., 2018). However, the misuse and overuse of antibiotics in treating bacterial infections such as TB resulted in the emergence of multidrug-resistant tuberculosis (MDR-TB), which is resistant to isoniazid and rifampicin (Caminero, 2005). Although the BCG vaccine is widely used as a preventive measure, it has several limitations, including variable efficacy across populations, ethnicities, and geographic regions. Moreover, the protective effect of BCG wanes over time, particularly in adulthood (Kumar, 2021).

While new TB vaccine candidates are currently undergoing clinical trials, there remains a pressing need for a more effective, long-lasting vaccine with broader population coverage and reduced risk (Rowland and McShane, 2011).

The emergence of the field of immunoinformatics, more specifically, the reverse vaccinology approach, aids in the development of the vaccine design process. Moreover, the successful application of several bioinformatic tools is considered advantageous compared to the traditional way of vaccine design (Reginald et al., 2018). Additionally, careful identification of an immunogenic protein antigen is crucial in vaccine design since it can be used for an *in silico* epitope prediction (Bahrami et al., 2019). Even though the MTb mutation rate is considered low (Eldholm and Balloux, 2016), the emergence of MDR-TB makes the TB infection challenging to eradicate. Epitope immune evasion can reduce the recognition of antigenic targets, compromising vaccine efficacy and potentially leading to vaccine escape (Coscolla et al., 2015). Hence, one of the best strategies to employ is the use of a reverse vaccinology approach in designing an immunotherapeutic agent that can prompt long-term immunity by identifying highly conserved epitopes in the sequences of essential immunogenic protein antigens in MTb. The application of immunoinformatics surmounts challenges on epitope immune evasion and costly vaccine development procedures as it is more specific and more cost-effective, with a faster workflow procedure than the traditional workflow involved in vaccine development (Bahrami et al., 2019; Reginald et al., 2018).

In this study, computational tools and databases were employed to analyze five MTb secretory proteins – ESAT-6, SpmT, CpnT, EspG, and PknG — all of which play critical roles in MTb’s phagolysosome inhibition and phagosome perforation (Pal et al., 2022; Upadhyay et al., 2018). These proteins also contribute to the transition from latent TB to active TB infection by disrupting granuloma integrity, leading to the release of dormant MTb and progression to active disease (Cambier et al., 2014). For instance, ESAT-6 has been implicated as a β2M binder and sequesters β2M in the endoplasmic reticulum. The binding action further inhibits the surface expression of the MHC-1:β2M complex, therefore impeding the presentation of peptides to CD8+ T cells (Sreejit et al., 2014). ESAT-6 also interacts with Toll-like receptor 2 (TLR2), inhibiting the production of key proinflammatory cytokines such as IL-12, TNF-α, and IL-6, ultimately preventing macrophages from eliminating phagocytosed MTb (Pathak et al., 2007). The SpmT secretory protein is involved in protein cleavage and maturation. Pal et al. (2022) reported that SpmT secretory protein is critical for MTb’s interaction with the host cell. As a serine protease, it helps ensure the proper functioning of MTb virulence factors via evasion of host immune response. Moreover, SpmT can degrade host immune effectors and modulate bacterial surface proteins to avoid immune detection, allowing MTb to persist inside macrophages (Upadhyay et al., 2018).

Another secretory protein, CpnT, is known to induce necroptosis by converting NAD+ into NAD and Radp, activating RIPK3 and inhibiting caspase-8, leading to ROS production. This process results in the necrotic cell death of macrophages, facilitating MTb dissemination (Sun et al., 2015).

PknG, a serine/threonine kinase, has been shown to inhibit phagosome–lysosome fusion, thereby enhancing MTb survival inside macrophages by targeting Rab7I1 (Walburger et al., 2004). Additionally, PknG suppresses pro-inflammatory cytokines such as IL-1β and IL-6, further promoting MTb survival through interactions with cyclophilin A (Wu et al., 2018).

Lastly, EspG disrupts host trafficking proteins involved in secretion and autophagy, such as GTPase Rab7, preventing phagosome–lysosome fusion and enabling MTb to persist in a non-acidic environment inside macrophages (Pal et al., 2022).

These five secretory proteins are crucial protein targets because of their roles in MTb’s virulence and pathogenicity, specifically for MTb’s ability to survive, replicate, and evade host immune response. By utilizing advanced computational and bioinformatics tools, this study successfully predicted CTL and HTL epitopes that have the potential to induce both cellular and humoral immunity, providing a promising foundation for future tuberculosis vaccine development (Yazdani et al., 2020).

Before conducting conservancy analysis, over 100 full-length sequences of the selected secretory proteins were retrieved from NCBI to ensure a comprehensive assessment of genetic diversity across different MTb strains and isolates. This approach enhances the reliability of identifying highly conserved epitopes, which are essential for eliciting long-term and broad immune responses across diverse MTb strains while mitigating epitope immune evasion (Criscuolo and Gribaldo, 2010). Lastly, it improves statistical confidence in identifying highly conserved peptide fragments, reducing the chance of sampling bias and increasing prediction accuracy. The conservancy analysis applied a Shannon entropy index ≤ 0.1, meaning that the identified peptide fragments, as summarized in the supplementary material, exhibited minimal sequence variability across different MTb strains (Liu and Bahar, 2012). The conservation of these sequences suggests that the predicted epitopes can trigger long-term immune responses in humans, a claim supported by studies demonstrating that highly conserved epitopes induce robust immunogenic responses and are essential for vaccine development against MTb (Martinez-Olivares et al., 2023; Ruaro-Moreno et al., 2023).

The cytotoxic T cells have been implicated in the direct killing and destruction of infected cells, where infected cells are directly recognized and lysed via pathogen-derived peptides. In contrast, the helper T cells help other immune cells, such as cytotoxic T cells, activate macrophages (Mishra, 2020). Consequently, the CTL can recognize antigenic peptides via the MHC class I antigen presentation, while the HTL uses the MHC class II in recognizing antigenic peptides on the surface of an infected target cell (Mishra, 2020; Turgeon, 2014). While CTL-mediated responses are crucial for containing intracellular pathogens, incorporating MHC class II-restricted epitopes that activate HTLs is invaluable for eliciting a more balanced and robust immune response (Habib et al., 2024). In particular, TH1 cells play a critical role in activating macrophages, the primary host cells of MTb (Zhuang et al., 2024), while also promoting cell-mediated immunity through CTL activation and macrophage-mediated bacterial killing (Maphasa et al., 2021). TH2 cells, on the other hand, facilitate B cell activation and antibody production, contributing to infection control and enhancing overall immunity (Stewart et al., 2023). By inducing both TH1 and TH2 responses, a vaccine that stimulates cell-mediated and humoral immunity can provide broader and more effective protection against TB (Chung et al., 2022).

The dual approach of using CTL and HTL-predicted epitopes from the same antigen that bind both MHC class I and MHC class II molecules offers several immunological benefits. It can enhance the efficacy and longevity of the immune response, particularly in the context of vaccine development. For instance, selecting epitopes from the same antigen that bind to both MHC class I and MHC class II molecules allows the vaccine to simultaneously activate both cytotoxic CD8+ T cells (via MHC class I) and helper CD4+ T cells (via MHC class II) (Kissick et al., 2014). The activation of CD8+ T cells is crucial for directly eliminating infected cells. In contrast, CD4+ T cells play a central role in orchestrating the broader immune response, including enhanced proliferation and activation of CD8+ T cells and activation of macrophages for improved pathogen clearance (Shepherd and McLaren, 2020).

The interplay between CD4+ and CD8+ T cells is well-established in generating an effective immune response (Swain et al., 2012). TH1 CD4+ T cells aid CD8+ T cells by secreting IL-2, a cytokine that promotes CD8+ T-cell proliferation and differentiation into CTLs (Laidlaw et al., 2016). Studies indicate that CD8+ T cells activated in the presence of CD4+ T cells exhibit enhanced functionality, persistence, and memory formation (Ahrends et al., 2019). Thus, incorporating epitopes that activate both MHC classes fosters a synergistic immune response, optimizing the generation of effective and long-lasting CD8+ T-cell responses.

A key objective of vaccination is to induce long-term immunological memory. A dual approach that stimulates both CD8+ and CD4+ T cells increases the likelihood of generating durable memory T cells. Memory CD8+ T cells provide rapid immune responses upon re-exposure to the pathogen, while memory CD4+ T cells are essential for immune surveillance and support of other immune cells (Raphael et al., 2020).

This study outlined a comprehensive approach for predicting CTL and HTL T-cell epitopes from the highly conserved sequences of five MTb secretory proteins. The identified candidate epitopes demonstrated promiscuous binding to the most common HLA class I and II alleles, suggesting broad population coverage. The large global, Southeast Asian, and Philippine population coverage, as estimated in the IEDB tool, may indicate wider population coverage in *in vivo* studies in the future. The high population coverage analysis is essential due to the occurrence of the MHC molecule’s polymorphic property that can affect the interaction between the MHC peptide binding groove and the predicted epitopes (Ebrahimi et al., 2019). Therefore, promiscuity is a crucial consideration in vaccine design. This is further validated by the study of Andongma et al. (2023), where they utilized highly promiscuous multiepitope for vaccine design against TB with enhanced immune efficacy. The strong binding affinity (MHC IC_50_ ≤ 500 nM) of candidate epitopes with the most common HLA alleles supports the claim of possessing wider human population coverage. Apart from the IC_50_ value generated for the CTL epitopes, other valuable data, such as TAP and proteasome scores, were considered. It has been well established that a positive TAP score indicates higher substrate affinity to TAP transporters or toward N-terminal prolonged regions, facilitating efficient transport to the endoplasmic reticulum (ER) (Abele and Tampe, 1999). Additionally, positive proteasome scores suggest favorable C-terminal cleavage during antigen processing, leading to increased peptide fragment availability for MHC class I molecules on the surface of infected cells (Fleri et al., 2017). In vaccine design, favorable MHC binding of predicted epitopes is as critical as ensuring their safety profile. Thus, this study excluded allergenic, cross-reactive, and toxic epitopes from the final CTL and HTL candidate selection. Using bioinformatics tools with standard parameters, the candidate epitopes were classified as potentially non-allergenic, non-cross-reactive, and nontoxic to humans. Additionally, the sequences of predicted epitopes did not significantly match (no 100% sequence similarity and no 100% sequence coverage) with any human proteome surveyed in the database, as supported by BLASTp analysis, resulting in E-values ranging from 3.3 to 85. Research suggests that peptide queries with *E*-values < 10^–6^ or 0 are considered cross-reactive, whereas *E*-values > 1 indicate non-cross-reactive peptides (Hileman et al., 2002; Pearson, 2013). These findings imply that the predicted CTL and HTL epitopes pose minimal risk of autoimmune concerns when administered in humans. The same consideration was applied by Andongma et al. (2023) to exclude allergenic and nonantigenic epitopes in the MTb vaccine design since antigenicity is required to effectively stimulate humoral immune responses, leading to memory cell generation against the epitopes of MTb. Moreover, hypersensitivity reactions in humans are a key consideration in vaccine development (Park et al., 2018); hence nonallergenic epitopes are deemed safe. The HLAepitope molecular docking analysis revealed dissociation constant (*K*_D_) values in the magnitude of 10^–12^ M or lower, indicating strong and favorable epitope binding within the peptide-binding groove of the HLA molecules. This is because the *K*_D_ value of a complex such as a peptide–epitope complex with more than 100 nM favors complex dissociation (Motulsky and Neubig, 2010). The epitope is not bound to the peptide binding groove of the HLA molecule. Additionally, negative Δ*G*, as summarized in Table 4 for all docked HLA–epitope complexes, supports the claim of spontaneous HLA–epitope complex formation (Galicia et al., 2023). The positive and spontaneous binding (negative Δ*G*) of the predicted epitopes indicates that they are more likely to be recognized as antigens and elicit robust immune responses. Additionally, the negative ΔG suggests the stability of the complex (epitope–HLA), indicating that the epitope will bind strongly and tightly to the peptide binding groove of the HLA alleles and may be considered a candidate for further experimental validation in terms of vaccine development against TB. Ghaffar et al. (2024) reported the same result where the HLA–epitope complex had a negative Δ*G* and low *K*_D_ values, indicating spontaneous binding and stable multiepitope vaccine design against *Haemophilus para-influenzae.*

The HLA–epitope interactions also contribute to complex formation’s overall spontaneity and favorability. Specifically, the hydrogen bonds promote higher affinity receptor–ligand molecular interactions by eliminating the interference of water molecules. This is because water molecules can compete with the epitope binding to the peptide binding groove of MHC molecules through hydrogen bond formation (Chen et al., 2016; Pace et al., 2014). Moreover, the strength of this intermolecular force of attraction (hydrogen bond) heavily relies on the donor-acceptor distances. It has been well established that a donor–acceptor distance ranging from 2.2 to 2.5 Å is considered “strong, mostly covalent,” while a distance range of about 2.5–3.2 Å is characterized as “moderate, mostly electrostatic.” Lastly, the 3.2–4.0 Å distance range is considered to have weak electrostatic interactions (Jeffrey, 1997). Thus, the donor-acceptor distance range of this study’s 10 representative HLA–epitope molecular interactions falls within the “strong, mostly covalent” category, as indicated by a donor-distance range of 2.0–2.5 Å. This does not mean that the interaction results in the formation of covalent bonds but indicates that the strength of intermolecular forces of attraction can be likened to the strength of the intramolecular covalent bond.

While this study leverages advanced *in silico* tools and algorithms, computational epitope predictions have inherent limitations (Oli et al., 2020). Although these methods are powerful for shortlisting candidate epitopes, they rely heavily on existing datasets and predictive algorithms, which can introduce biases (Peters et al., 2020). The accuracy of predicted epitopes is also contingent on the completeness and representativeness of protein databases, which may lack sufficient genetic diversity for certain populations and pathogens (Sidney et al., 2020).

Furthermore, the candidate epitopes identified in this study have not yet been validated in clinical trials, which remains the ultimate experimental benchmark for vaccine development. To support the specific workflow developed in this research, future *in vitro* and *in vivo* assays are anticipated. *In vitro* validation can include MHC binding affinity assays using proteolyticmass spectrometry, surface plasmon resonance (SPR) biosensor analysis, or enzyme-linked immunosorbent assay (ELISA) to confirm predicted epitope–HLA interactions (Jiang et al., 2023; Rinaldi et al., 2019). Additionally, T-cell activation assays, such as interferon-gamma release assays, can assess the ability of predicted epitopes to stimulate immune responses.

Beyond *in vitro* analyses, *in vivo,* experiments using animal models, such as mouse models of tuberculosis, can be employed to evaluate CTL and HTL responses and analyze memory T cell generation, which is essential for long-term immunity (Coppola et al., 2021; Chugh et al., 2024). Without complementary experimental validation, the potential of immunoinformatics-predicted epitopes remains constrained. Nevertheless, this study establishes a strong and novel foundation for identifying promising vaccine candidates, warranting further *in vitro* and *in vivo* experimentation and, ultimately, clinical trials.

## Conclusions

This research presented strategic identification of novel CTL and HTL epitopes from secretory antigens that could be incorporated into future vaccine formulations against MTb. The predicted epitopes were derived from highly conserved sequences of five key secretory proteins involved in phagosome maturation disruption. Docking data from the study, supported by favorable and spontaneous measures, indicate that the candidate CTL and HTL epitopes can potentially elicit immunogenicity against target secretory proteins highly expressed in MTb. Lastly, all candidate epitopes demonstrated an acceptable safety profile, showing no allergenic, toxic, or cross-reactive properties, making them suitable for future immunotherapeutic applications. However, further *in vitro* and *in vivo* validation is required to confirm the immunogenicity of these candidate epitopes and assess the consistency of the computational prediction models used in this study.

## Supplementary material

The detailed information regarding the predicted CTL and HTL epitopes from the sequences of the secretory proteins can be accessed and downloaded through this link: CD4+ PREDICTED EPITOPES SUMMARY.xlsx and CD8+ PREDICTED EPITOPES SUMMARY.xlsx.
